# Facial phenotyping of Holstein calves using pose estimation models with varying keypoints

**DOI:** 10.3168/jdsc.2025-0923

**Published:** 2026-03-06

**Authors:** Camila S. Mussi, Amy R. Reibman, Jacquelyn P. Boerman, Lauryn R. Unversaw, Gabriel C. Medeiros, José Bento S. Ferraz, Elisangela C. Mattos, Isabela G.M.A. Santos, Luiz F. Brito

**Affiliations:** 1 Department of Animal Sciences, Purdue University, West Lafayette, IN 47907; 2 Department of Veterinary Medicine, College of Animal Sciences and Food Engineering, University of São Paulo, Pirassununga, SP, 13635-900, Brazil; 3 Elmore Family School of Electrical and Computer Engineering, Purdue University, West Lafayette, IN 47906

## Abstract

Artificial intelligence is increasingly applied in livestock farming and offers valuable tools for monitoring animal health and welfare. Among these, facial expressions have emerged as informative indicators of well-being in animals. In this context, the main objective of this study was to evaluate the accuracy of pose estimation models for extracting facial features in North American Holstein calves. Three YOLOv8-based models were trained using 10, 16, and 30 facial keypoints, and their performance was evaluated using training metrics (loss, precision, recall, F1 score, training time, and number of epochs) and keypoint accuracy measures (object keypoint similarity, average precision, average recall). The 10-keypoint model outperformed the others, requiring less training time, achieving lower loss, and yielding the highest prediction accuracy. This model enabled reliable extraction of facial phenotypes, including nostril distance, eye opening, and ear angles and movements. These findings demonstrate the potential of pose estimation models for automated phenotyping and welfare monitoring in dairy calves.

## Highlights

• Pose estimation enables automated phenotyping and welfare monitoring in calves.
• Fewer keypoints may improve model efficiency with limited training data.
• Facial traits, including nostril distance and eye opening, were accurately extracted.
• Ear position and movement were accurately quantified by the model.

## Graphical Abstract

**Figure:**
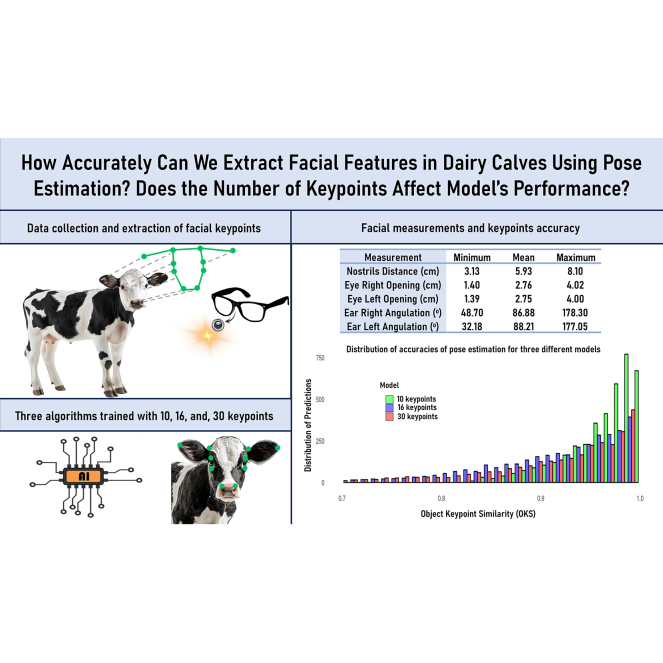
**Summary:** This study evaluated the accuracy of facial feature extraction in dairy calves using pose estimation models, with a focus on the effect of keypoint quantity. Three YOLOv8-based models were trained with 10, 16, and 30 facial keypoints. Model performance was assessed using various training metrics (loss, precision, recall, training time, and epochs) and keypoint prediction accuracy metrics, including object keypoint similarity, average precision, and average recall. Increasing the number of keypoints reduced prediction accuracy and overall model performance. The 10-keypoint model yielded the best results and enabled reliable extraction of relevant phenotypes, such as ear angles and movements, nostril distance, and eye opening.

Digital technologies and artificial intelligence (**AI**) are increasingly being adopted on livestock farms, including dairy operations, to optimize management and decision-making (Buller et al., 2020; Groher et al., 2020). These technologies enable monitoring of growth patterns, behavior, early signs of diseases, welfare, and feeding habits, providing valuable data that can be leveraged to improve animal welfare and performance (Neethirajan and Kemp, 2021; Bao and Xie, 2022). Facial movements and expressions are widely used to assess emotions, reactions, and overall welfare in humans (Martinez and Du, 2012) and have also been investigated in other animals (e.g., Keeling, 2019; Neethirajan, 2021). Facial features represent noninvasive indicators of health and welfare (Papageorgiou and Simitzis, 2022). In cattle, commonly studied indicators include ear movements and positions (Proctor and Carder, 2014; Machado et al., 2024) and eye-related traits such as visible sclera and eye opening (Proctor and Carder, 2015; Gómez et al., 2018). However, most studies have focused on adult cows, whereas research on calves examining health and behavior through facial features remains limited.

Health and welfare indicators in cattle are commonly based on posture or locomotion, such as movement patterns, and have proven valuable for detecting illness and lameness (Sadiq et al., 2017). Facial traits provide an additional source of information by capturing subtle phenotypic expressions that may reflect pain, stress, or discomfort (Proctor and Carder, 2015; Gómez et al., 2018). Changes in facial features, such as ear position or facial action patterns, may precede overt clinical signs or measurable activity changes, and may enable earlier detection of stress or illness in a noninvasive and scalable manner suitable for on-farm applications. Beyond health and welfare monitoring, these facial traits may also be novel auxiliary digital phenotypes associated with other complex traits, including temperament and behavior, offering additional opportunities for integration into genetic and genomic studies.

Pose estimation is a valuable approach for identifying specific point locations in images and videos. Object detection algorithms such as you only look once (**YOLO**), widely known for their efficiency in object detection (Jiang et al., 2022; Bao, 2024), can enhance the precision of pose estimation. The version YOLOv8 (Jocher et al., 2023), developed by Ultralytics, balances precision and speed in object detection (Hussain, 2023; Terven et al., 2023). In this regard, the main objective with this study was to perform facial phenotyping in dairy calves by applying keypoint detection to features such as ear position and movement, eye opening, and nostril distance. Three YOLOv8l-pose models trained with different numbers of keypoints (10, 16, and 30) were compared in terms of training performance and keypoint accuracy. The primary objective was to determine how accurately features related to animal health and welfare could be predicted and to identify the most effective model. A secondary objective was to extract and postprocess phenotypes based on the model's predictions of facial features.

Data collection was conducted on the Purdue University's dairy farm (West Lafayette, IN), where 67 Holstein calves were recorded at least once during routine husbandry activities. Video recordings were performed weekly from September to December 2024, capturing all calves present on each recording day. Some animals were recorded on different days, and new calves were added progressively according to their birth. The animals were housed individually under standard farm conditions, with no special interventions. Calves were born between August 18 and December 11, 2024. All videos were recorded using iVUE RoHS HD 1080P camera glasses (iVUE Camera). In total, 70 videos were obtained from 67 calves. These videos included more than one animal each and were subsequently split by individual calves per day recorded, which resulted in 269 videos. These videos were converted into frames at a rate of 1 frame per second and manually filtered according to the criterion that the calf had to be facing the camera with the entire head clearly visible. This process resulted in a final dataset of 6,702 high-quality images. From the complete dataset, 10% of the images (n = 670) were selected for training, whereas the remaining 90% were retained for prediction and subsequent analyses.

All image labeling for training preparation, including bounding boxes and keypoints, was performed using the Roboflow platform (https://roboflow.com). Keypoints were selected based on anatomical regions relevant for extracting facial measurements, with preference given to landmarks that were visually clear and easy for the model to detect, whereas central or ambiguous areas were avoided. The keypoint annotations were cumulative: The 10-point model included a core set of locations, which were expanded to 16 points and further extended to 30 points. The progressive addition of landmarks was intended to enable the extraction of increasingly complex phenotypic traits, with the initial 10 keypoints supporting basic measurements, the 16-keypoint configuration providing additional anatomical details, and the 30-keypoint model allowing for the potential derivation of more refined and complex traits. To minimize human error and ensure consistency, all annotations were performed by a single annotator. The final set of keypoints is illustrated in Figure 1.

**Figure 1:**
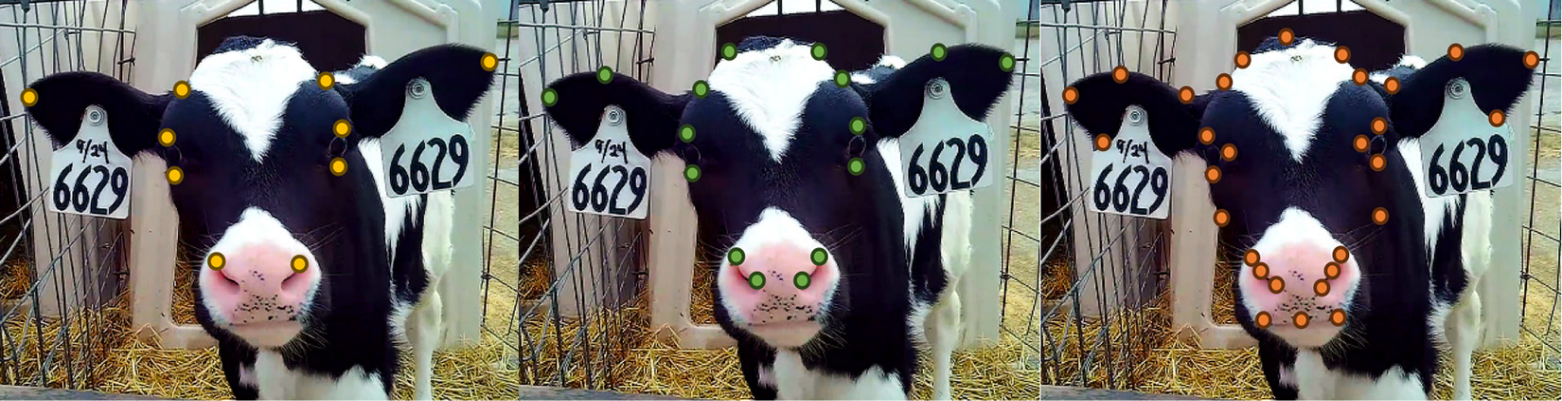
Examples of facial annotations using 10, 16, and 30 keypoints.

The annotated dataset was divided into training, validation, and testing sets using an animal and image ID-based split, ensuring that no animals and images were duplicated across subsets. In total, 70% of the images were used for training, 15% for validation, and 15% for testing. Preprocessing steps included automatic orientation adjustments to improve model recognition across different angles and resizing images to a standardized resolution of 640 × 640 pixels. Data augmentation techniques were also applied, including a 10% increase in brightness to enhance model robustness. Pose estimation was performed using the large variant of the YOLOv8 pose estimation model, selected based on preliminary testing and guidance from the Ultralytics documentation. Model training and inference were performed on a GPU-enabled machine (NVIDIA-SMI, Driver Version: 550.120, NVIDIA Corporation). All 3 models were trained for up to 1,000 epochs, with 1 epoch corresponding to a full pass through the training set. Early stopping was applied when validation loss failed to improve for 100 consecutive epochs, indicating convergence. Model predictions were conducted on the same machine to ensure consistency. Model evaluation was performed in 2 main stages: (1) right after training, by analyzing the metrics generated for each training session, and (2) after prediction, by analyzing keypoint detection accuracy across the 3 models.

The first evaluation stage assessed overall training performance by examining metrics such as training duration and the number of iterations required for convergence. For each model, the best-performing epoch was identified, and key performance metrics (loss, precision, and recall) were extracted. The F1 score, calculated as the harmonic mean of precision and recall, was used for balanced performance evaluation. Subsequently, the models were evaluated for keypoint detection accuracy using the object keypoint similarity (**OKS**) metric, which quantifies the similarity between predicted and true keypoints while accounting for object scale, measured by the object area in pixels, and keypoint visibility (Ruggero Ronchi and Perona, 2017). The OKS values range from 0 (no similarity) to 1 (perfect keypoint detection). For consistency in comparison across models, only the 10 keypoints common to all 3 models were analyzed. The OKS results were assessed across predefined thresholds ranging from 0.50 to 0.95 (Ruggero Ronchi and Perona, 2017), where predictions with OKS scores within the threshold were classified as true positives, whereas those falling below were considered false positives. From these classifications, we computed the average precision (**AP**) and average recall (**AR**). All results obtained are summarized in Table 1.

**Table 1:** Evaluation metrics for all 3 models (10, 16, and 30 keypoints) based on the best trained model during the epochs^1^

Model	Time (h)	Epoch	Loss	F1	AP_OKS_	${\mathrm{AP}}_{\mathrm{OKS}}^{0.75}$	${\mathrm{AP}}_{\mathrm{OKS}}^{0.95}$	AR_OKS_	${\mathrm{AR}}_{\mathrm{OKS}}^{0.75}$	${\mathrm{AR}}_{\mathrm{OKS}}^{0.95}$
10	2.390	529	0.156	0.958	0.981	0.973	0.981	0.954	0.983	0.985
16	3.703	818	0.521	0.958	0.961	0.928	0.948	0.812	0.752	0.837
30	4.199	913	2.054	0.943	0.895	0.915	0.911	0.798	0.830	0.865

1 AP_OKS_ = average precision based on OKS values; ${\mathrm{AP}}_{\mathrm{OKS}}^{0.75}$ = average precision at 0.75 based on OKS values; ${\mathrm{AP}}_{\mathrm{OKS}}^{0.95}$ = average precision at 0.95 based on OKS values; AR_OKS_ = average recall based on OKS values; ${\mathrm{AR}}_{\mathrm{OKS}}^{0.75}$ = average recall at 0.75 based on OKS values; ${\mathrm{AR}}_{\mathrm{OKS}}^{0.95}$ = average recall at 0.95 based on OKS values.

Following the evaluation, the 10-keypoint model was used to generate predictions and extract phenotypic measures from the remaining 90% of the dataset (6,032 images), which were retained exclusively for prediction analyses. Postprocessing was applied to derive behavior- and welfare-related traits, including ear position and the time spent in each position, number of times moving the ears, eye opening, and nostril distance. Ear position was determined through ear angulation, calculated using 3 keypoints: the ear tip, the ear attachment point, and a point above the eye. Angles were computed by taking the Euclidean distance (Wang et al., 2005) between the points and the angle formed on the head keypoint was calculated using the law of cosines. An ear was classified as in backward position when the angle exceeded 90° and frontward position when the angle was below or equal to 90°. The time each ear spent in backward or forward positions was calculated based on the frame rate and ear angle, with results reported separately for the left and right ears. Ear movement was quantified only when 20 consecutive frames (20 s) were available, with each transition between forward and backward positions counted as a movement. Eye opening was measured as the Euclidean distance between 2 keypoints placed above and below the eye, and nostril distance was calculated in the same way between the 2 keypoints at the nostrils. Distances in pixels were converted into centimeters based on a tool that recognized the animal's ear tag, which has a known size, thereby allowing scaling of the measurements. A descriptive summary of these extracted phenotypes is provided in Table 2.

**Table 2:** Facial metrics obtained through postprocessing of predicted keypoints

Item	Minimum	Mean	Maximum	SD	CV (%)
Nostril distance (cm)	3.13	5.93	8.10	0.82	14
Eye right opening (cm)	1.40	2.76	4.02	0.40	14
Eye left opening (cm)	1.39	2.75	4.00	0.46	16
Time ear front position (s)	2.00	9.72	20.00	1.15	11
Time ear back position (s)	0.00	5.43	16.00	1.05	19
Ear movement (no. of times)	0.00	3.73	9.00	0.79	21

Evaluating model performance during training is essential for understanding learning efficiency and enabling comparisons across models. All 3 models successfully tracked facial keypoints in calf images, although their performance differed (Table 1). Models with 10, 16, and 30 keypoints were initially assessed to determine whether increased keypoint density could support the extraction of more detailed phenotypic traits. However, given the available dataset, models with fewer keypoints provided more reliable predictions. The 10-keypoint model achieved the shortest training duration and lowest loss, whereas the training time and the number of epochs increased with the number of keypoints, reflecting greater complexity of models with more landmarks. The F1 scores, which balance precision and recall, were high across all models (0.943 to 0.958), indicating accurate predictions with few false positives. Loss values ranged from 0.156 in the 10-keypoint model to 2.054 in the 30-keypoint model, confirming greater performance of the simpler model. Considering a dataset of 670, the 10-point model outperformed the others, suggesting that models with fewer keypoints can be more precise, faster, and more computationally efficient when trained on relatively small datasets.

The OKS approach provides a robust measure of keypoint accuracy by accounting for factors such as landmark visibility, object size, and the relative difficulty of detecting specific keypoints (Ruggero Rochi and Perona, 2017). Previous studies have shown that OKS is a reliable tool for evaluating pose estimation performance (Maji et al., 2022; Saleh et al., 2023). Based on OKS values, AP and AR are commonly used to assess model performance (Ali et al., 2024), relying on the classification of predictions as true or false positives across varying thresholds; AP measures the accuracy of positive predictions, whereas AR quantifies the ability of the model to correctly detect keypoints, thus providing complementary perspectives on performance (Padilla et al., 2020). In the present study, the 10-point model achieved the highest AP (0.981), compared with 0.961 for the 16-point model and 0.895 for the 30-point model, indicating superior precision. At fixed OKS thresholds, the 10-point model again outperformed the others, with AP values of 0.973 (OKS: 0.75) and 0.981 (OKS: 0.95), compared with 0.928 to 0.948 and 0.911 to 0.915 for the more complex models. For the AR values (across OKS thresholds from 0.50 to 0.95), the 10-point model proved superior results, achieving an AR of 0.954 compared with 0.812 for the 16-point model and 0.798 for the 30-point model. At fixed thresholds, the 10-point model reached 0.983 (OKS 0.75) and 0.985 (OKS: 0.95), consistently higher than the more complex models. These results indicate that the 10-point model consistently achieved the highest proportion of true positive predictions across all thresholds, demonstrating superior recall in keypoint detection. Taken together with AP results, the 10-point model consistently outperformed the 16- and 30-point models, suggesting that models with fewer keypoints may yield more accurate detections when trained on the same dataset. This outcome also indicates that increasing the number and complexity of landmarks may require larger training datasets to reliably learn all keypoints, potentially affecting performance across both basic and complex landmarks.

As shown in Table 2, the extracted features provide meaningful insights into calf health and welfare. These results also highlight the potential for defining novel phenotypes that may be relevant for future research and potentially serve as auxiliary traits in breeding programs. Most existing studies on facial expressions focus on adult dairy cows, and relatively little is known about their relevance in calves. As behavior and facial expression may change throughout an animal's development, interpretations of facial indicators in young animals may differ from those in adults, underscoring the importance of calf-specific analyses. In this context, facial structures, alongside other physical parameters, can serve as reliable, noninvasive indicators of health and overall well-being in calves (Lowe et al., 2019; Tschoner, 2021).

In adult animals, ear postures and the frequency of ear movements have been investigated as potential indicators of emotional states (Papageorgiou and Simitzis, 2022). Using the 10-keypoint model, we successfully tracked ear-related keypoints and, after postprocessing, extracted several traits (Table 2). On average (± SD), calves held their ears in the forward position for 9.72 ± 1.15 s and in the backward position for 5.43 ± 1.05 s. Ear movement, defined as the number of transitions between forward and backward positions, averaged 3.73 ± 0.79. Previous studies in adult cows have shown that ear posture and movement are influenced by ongoing experiences (Proctor and Carder, 2014; Ginger et al., 2023). For instance, adult cows are generally considered to be relaxed when their ears move less frequently and remain in a position that falls perpendicularly to the head, indicating reduced muscular tension (Proctor and Carder, 2014). In our study, the animals were removed from their housing, and their attention was held by the person recording the video, which likely kept them curious or anxious, sometimes expecting to be fed. Therefore, our results do not fully represent calves in a completely relaxed state, although no comparable studies are currently available for direct reference.

Studies on sheep, cattle, and pigs have demonstrated associations between pain and changes in eye shape or nostril dilation (e.g., Gleerup et al., 2015; Neethirajan, 2021). In calves, dilated nostrils, tension above the eyes, partially closed eyelids, and increased muscle tone in the lips can indicate pain (Tschoner, 2021). In our study, the 10-point model successfully tracked keypoints that enabled the measurement of eye opening and the distance between the nostrils in centimeters. The mean eye opening was 2.76 ± 0.40 cm for the right eye and 2.75 ± 0.46 cm for the left eye, whereas the mean distance between the nostrils was 5.93 ± 0.82 cm. Although these measurements are influenced by head size and would benefit from longer observation periods to capture dynamic changes, our results demonstrate that combining keypoint detection with postprocessing can yield accurate and objective measurements. Given the relevance of these facial features, it is important to study them in calves. Incorporating traits related to stress response and resilience traits can contribute to healthier and more productive herds. Pose estimation models capable of identifying signs of stress or discomfort could support the genetic selection of animals that are less prone to stress, ultimately improving animal welfare and reducing management costs.

This study also had some limitations. The use of moving cameras likely introduced noise into the dataset, including image shaking and variations in angle, which combined with natural calf movements may reduce the model stability when applied to video data. To minimize these effects, analyses were performed on still frame images rather than full video sequences. For future studies, the use of fixed cameras positioned consistently at the level of the calf's head is recommended to improve dataset quality and model performance. In addition, further automation of the processing pipeline could reduce subjectivity and increase the reliability and reproducibility of the extracted phenotypes. Another limitation of this study was the relatively small annotated training set (670 images), which may have constrained the performance of more complex models, particularly the 30-keypoint configuration. Models with higher keypoint density introduce additional landmarks that are often more difficult to detect due to increased anatomical variability, partial occlusion, or lower image quality, and typically require larger datasets to be effectively learned. Within the constraints of the available data, the 10-keypoint model provided the most reliable and consistent predictions for the phenotypic traits evaluated. Future studies using larger annotated datasets may better exploit the potential of higher-density keypoint models.

In conclusion, this study establishes a novel approach to facial phenotyping in calves using AI. Pose estimation models proved effective for detecting early indicators of both positive and negative stimulus responses. Among the models tested, the 10-keypoint version outperformed the 16- and 30-keypoint models in both performance and OKS-based accuracy. Importantly, this model enabled reliable extraction of behavior- and welfare-related phenotypes, including ear posture and movement, eye opening, and nostril distance. These findings demonstrate the feasibility of using pose estimation for automated, noninvasive monitoring of calf welfare and highlight its potential for predicting behavioral and physiological responses in young animals. Logical next steps include the association of the derived traits with direct health and welfare indicators and estimation of their genetic background (e.g., heritability, repeatability, and genetic correlations with other relevant traits) using larger datasets.
